# Actuating Mechanism and Design of a Cylindrical Traveling Wave Ultrasonic Motor Using Cantilever Type Composite Transducer

**DOI:** 10.1371/journal.pone.0010020

**Published:** 2010-04-02

**Authors:** Yingxiang Liu, Weishan Chen, Junkao Liu, Shengjun Shi

**Affiliations:** State Key Laboratory of Robotics and System, Harbin Institute of Technology, Harbin, China; German Cancer Research Center, Germany

## Abstract

**Background:**

Ultrasonic motors (USM) are based on the concept of driving the rotor by a mechanical vibration excited on the stator via piezoelectric effect. USM exhibit merits such as simple structure, quick response, quiet operation, self-locking when power off, nonelectromagnetic radiation and higher position accuracy.

**Principal Findings:**

A cylindrical type traveling wave ultrasonic motor using cantilever type composite transducer was proposed in this paper. There are two cantilevers on the outside surface of cylinder, four longitudinal PZT ceramics are set between the cantilevers, and four bending PZT ceramics are set on each outside surface of cantilevers. Two degenerate flexural vibration modes spatially and temporally orthogonal to each other in the cylinder are excited by the composite transducer. In this new design, a single transducer can excite a flexural traveling wave in the cylinder. Thus, elliptical motions are achieved on the teeth. The actuating mechanism of proposed motor was analyzed. The stator was designed with FEM. The two vibration modes of stator were degenerated. Transient analysis was developed to gain the vibration characteristic of stator, and results indicate the motion trajectories of nodes on the teeth are nearly ellipses.

**Conclusions:**

The study results verify the feasibility of the proposed design. The wave excited in the cylinder isn't an ideal traveling wave, and the vibration amplitudes are inconsistent. The distortion of traveling wave is generated by the deformation of bending vibration mode of cylinder, which is caused by the coupling effect between the cylinder and transducer. Analysis results also prove that the objective motions of nodes on the teeth are three-dimensional vibrations. But, the vibration in axial direction is minute compared with the vibrations in circumferential and radial direction. The results of this paper can guide the development of this new type of motor.

## Introduction

Ultrasonic motors (USM) are a new type actuator which is based on the concept of driving the rotor by a mechanical vibration excited on the stator via piezoelectric effect. Their fundamental working principle consists in generating a mechanical vibration in the stator able to create an elliptical motion at the interface with the rotor. The rotor is in contact with the stator, and the driving force is the frictional force between rotor and stator. Ultrasonic motors exhibit merits such as simple structure, quick response, quiet operation, self-locking when power off, nonelectromagnetic radiation and higher position accuracy [1], [2].

According to the PZT working mode, USM can be classified into bonded type motors [3], [4] and bolt-clamped type motors [5], [6] up to the present. Theoretically speaking, the latter ones exhibit higher output power and efficiency than former ones by adopting d_33_ working mode of PZT, which have higher transfer efficiency than d_31_ mode used by bonded type motors. Thus, new configuration of USM adopted d_33_ working mode of PZT is the focus of this study.

In a previous work, a cylindrical standing wave USM using cantilever type longitudinal transducers was proposed and analyzed [7]. The simulation results verify the feasibility of excitation of a standing wave in a cylinder by using cantilever type longitudinal transducers. However, it's difficult to realize the reversing motion.

In this study, a cylindrical type traveling wave ultrasonic motor using cantilever type composite transducer is proposed, where a single transducer can excite a flexural traveling wave in the cylinder. The actuating mechanism of proposed motor is analyzed. The motor is designed and analyzed with FEM.

## Materials and Methods

### 1. Structure of the USM

**Figure 1:**
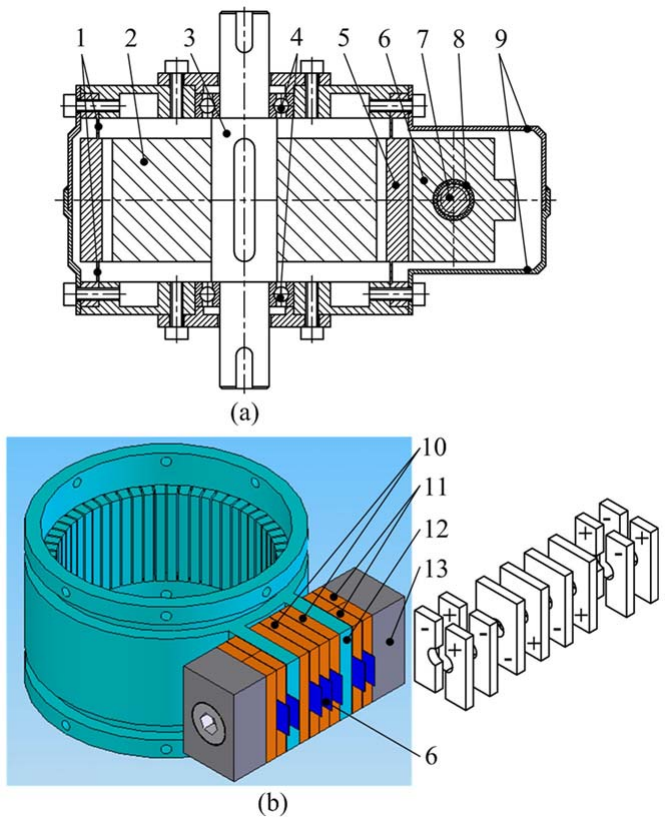
Structure of the USM (1-thin ring, 2-rotor mechanism, 3-output shaft, 4-bearing, 5-cylinder, 6-electrode, 7-screw, 8-insulation covering, 9-encloser, 10-longitudinal PZT, 11-bending PZT, 12-cantilever, 13-end cap). (a) Section view of proposed USM, (b) Three-dimensional model of stator.

Fig. 1(a) shows the section view of proposed USM, and Fig. 1(b) shows the three-dimensional model of stator and the polarization of PZT ceramics. The stator contains one cylinder and one cantilever type composite transducer, and the transducer locates on the outer surface of the cylinder. There are evenly distributed teeth on the inner surface of cylinder. Two cantilevers are located on the outside surface of cylinder, four longitudinal PZT ceramics are set between the cantilevers, and four bending PZT ceramics are set on each outside surface of cantilevers. A screw fastens the longitudinal PZT, bending PZT and end caps together to form the transducer. Beryllium bronze sheets are clamped to serve as electrodes. An expansion mechanism, not introduced in this study, is used to adjust preload between the rotor and stator. The proposed USM belongs to the bolt-clamped type motor, and the PZT ceramics work with d_33_ mode, which has high electromechanical coupling efficiency and can improve the mechanical output characteristics.

### 2. Actuating Mechanism

In order to excite a traveling wave in the cylinder, two standing waves, the amplitudes of which are equal, and their phase difference on time and space is π/2, are generated by the longitudinal and bending vibration of transducer, respectively. The longitudinal vibration of transducer is excited by the longitudinal vibration of longitudinal PZT. The bending PZT are located at the antinodal plane of bend wave of transducer, and the bending vibration of transducer is excited by the longitudinal vibration of bending PZT. Thus, the composite transducer should be excited with two-phase alternating voltages. When a flexural traveling wave is excited in the cylinder, elliptical trajectories are achieved at the particles on the teeth. And the driving force is the frictional force between the rotor and teeth.

**Figure 2:**
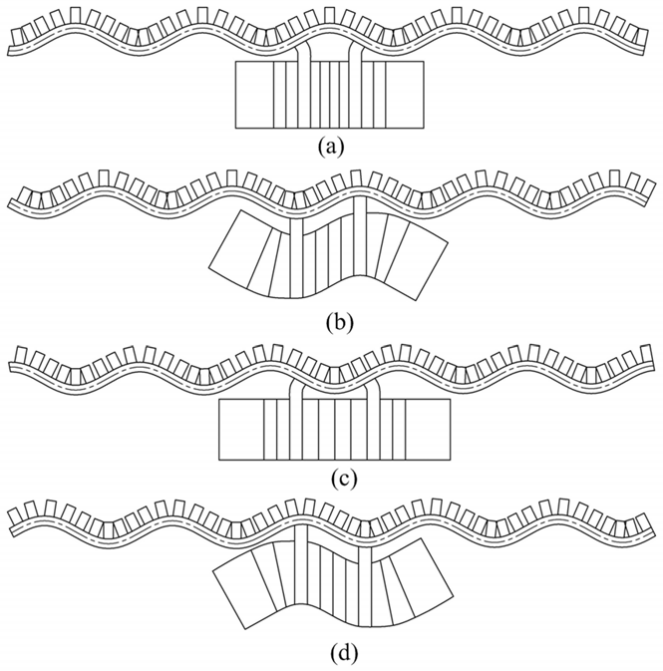
Vibration mode shapes of stator in one vibration cycle.

Fig. 2 shows the vibration mode shapes of stator in one vibration cycle (a development view in circumferential direction). Fig. 2(a) and Fig. 2(c) show the standing wave excited by the longitudinal vibration of transducer. Fig. 2(b) and Fig. 2(d) show the standing wave excited by the bending vibration of transducer. From Fig. 2(a) to Fig. 2(d), a flexural rightward traveling wave is excited in the cylinder. The direction of traveling wave can be changed by adjusting the phase difference of excitation voltages on time, which results in the reversing motion of rotor.

### 3. Design and Analysis

The actuating mechanism of proposed motor indicates that the key to form a traveling wave in the cylinder is the degeneration between the vibration modes of cylinder and transducer. FEM was used in this study to accomplish this work, and ANSYS software was used.

To realize the degeneration, a cylinder was designed, firstly. Then, a composite transducer with near resonance frequency was designed. The structure parameters of transducer were adjusted to make the resonance frequencies of longitudinal and bending vibrations are close with each other. And a preferential parameter for adjustment during degeneration was gained by the modal analysis. Finally, the finite element model of stator was founded. And the preferential parameter of transducer was adjusted to realize the degeneration between the resonance frequencies of two vibration modes of stator.

**Figure 3:**
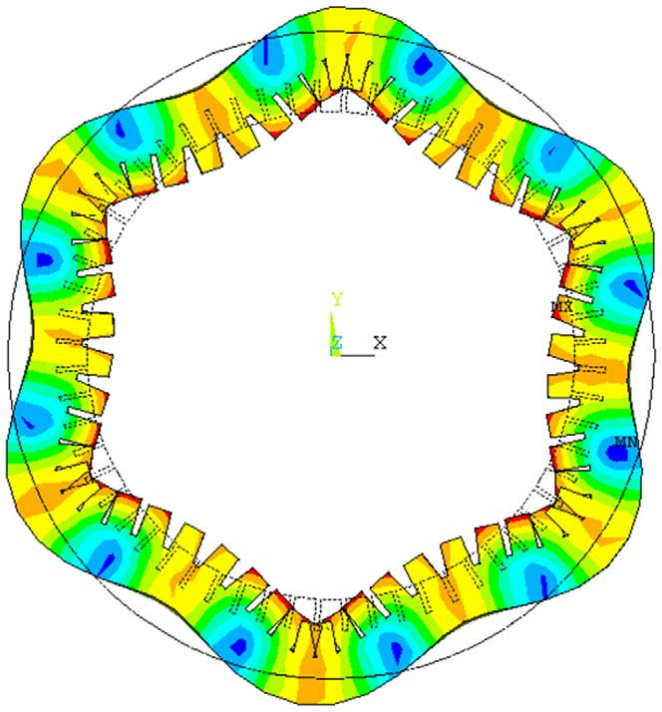
Vibration mode shape of cylinder.

Take B(*m*,*n*) to describe the bending vibration modal of cylinder, where *m* and *n* indicate nodal circles and diameters, respectively. B(0,6) was selected in this study for a suitable working frequency. Duralumin alloy (Mass density *ρ* = 2810 kg/m^3^, Young modulus *E* = 7.2×10^10^ N/m^2^, Poisson ratio *σ* = 0.33) is the selected material of cylinder and cantilevers. Fig. 3 shows the vibration mode shape of cylinder.

Set *D* as the outer diameter of cylinder, *d* as the inner diameter of cylinder, *H_c_* as the height of cylinder, *H_t_* as the height of teeth, *θ* as the span of teeth, *N* as the number of teeth. Table 1 shows the structural parameters of cylinder. The resonance frequency of B(0,6) vibration modal is 26.024 kHz. In a previous study, the design parameters sensitivity of the bending resonance frequency of cylinder was gained by using modal analysis, and results indicate that *D* and *d* have the most obvious effect on the resonance frequency [7].

**Table 1:** Structural parameters of cylinder.

*D*(mm)	*d*(mm)	*H_c_*(mm)	*H_t_*(mm)	*θ*(deg)	*N*
70.2	82.6	20	4	5	60

**Figure 4:**
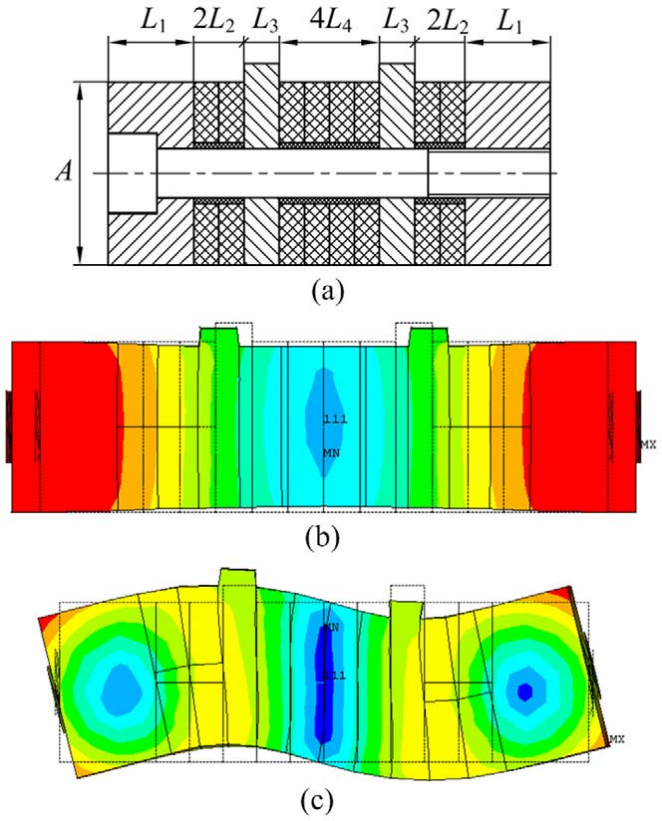
Section view and vibration mode shapes of transducer. (a) Section view of transducer, (b) Longitudinal vibration, (c) Bending vibration.

Fig. 4 is the section view and vibration mode shapes of the composite transducer, and the main structural parameters were listed. Steel (*ρ* = 7800 kg/m^3^, *E* = 2.06×10^11^ N/m^2^, *σ* = 0.3) is the selected material of end caps and screw. The PZT ceramics material is PZT-41.

Table 2 shows the initial structural parameters of transducer (*B* indicate the length of transducer in axial direction of cylinder). Modal analysis was developed, and results show that the resonance frequencies of the longitudinal and bending vibrations of transducer are 25.743 kHz and 26.257 kHz, respectively. During the modal analysis, SOLID227 element was used for the meshing, and the voltages applied on the nodes of electrodes were set as zero. Block Lanczos method was adopted to extract the vibration mode shapes and the resonant frequencies. The electrodes and glue lines were ignored, and the error is acceptable because the thicknesses of electrode and glue line are very small. Modal analysis results also show that *A* has obvious effect on the bending resonance frequency but has minor effect on the longitudinal resonance frequency. Thus, *A* should be the preferential parameter for adjustment during degeneration.

**Table 2:** Initial structural parameters of transducer (mm).

*L*_1_	*L*_2_	*L*_3_	*L*_4_	*A*	*B*
11.5	4	4	4	20	20

**Figure 5:**
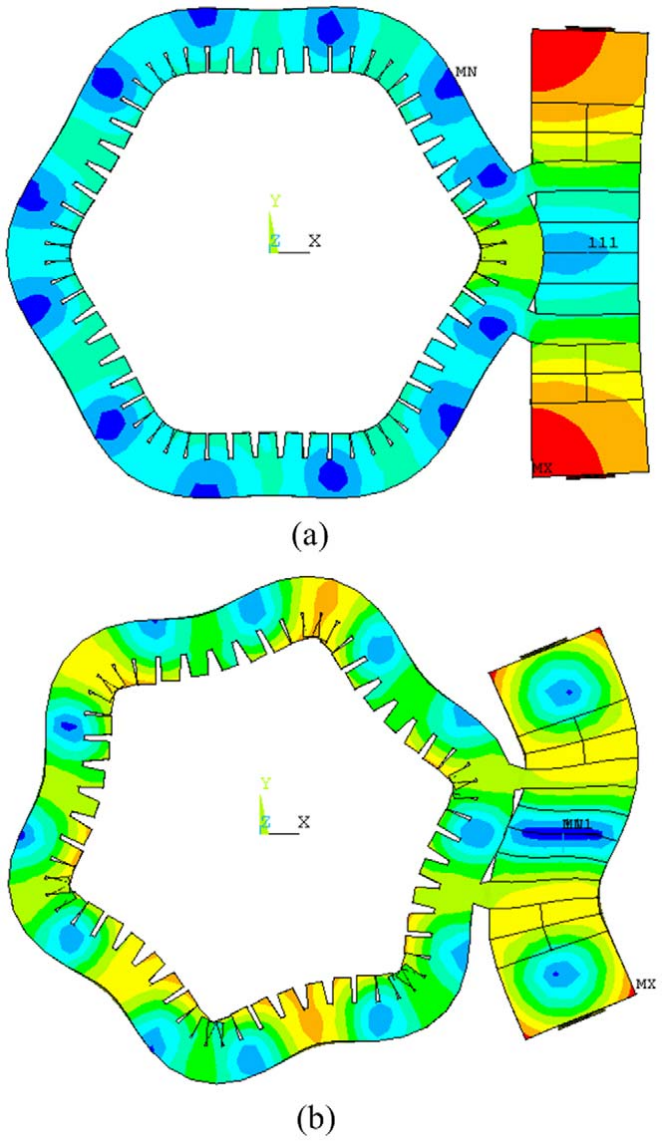
Vibration mode shapes of stator. (a) Standing wave excited by the longitudinal vibration of transducer, (b) Standing wave excited by the bending vibration of transducer.

The finite element model of stator was founded. Modal analysis was developed to extract the vibration modes of stator. Fig. 5 shows the two vibration modes of stator. In Fig. 5, two standing waves with six nodal diameters were excited in the cylinder by the longitudinal and bending vibrations of transducer, respectively. The phase difference of two standing waves on space is π/2, which is a necessary condition for a flexural traveling wave. The degeneration of resonance frequencies of two vibration modes of stator is another necessary condition. Based on the analysis results of cylinder and transducer, *A* was adjusted for the degeneration. Finally, when *A* = 18.6 mm the resonance frequencies of two vibration modes of stator degenerated at 26.276 kHz. The resonance frequencies of stator and cylinder are close to each other, which indicates the good degeneration between the transducer and cylinder.

According to the final structural parameters, the finite element model of stator was founded. Two-phase alternating voltages (the frequency is 26.276 kHz, the phase difference on time is π/2, the period number is 40, set *V_L_* as the virtual value of voltage applied on the longitudinal PZT, and *V_B_* as the virtual value of voltage applied on the bending PZT) were applied on the PZT ceramics to accomplish the transient analysis. As the amplitudes of two bending standing waves are equal is another necessary condition for a flexural traveling wave, rational value of voltages should be selected. First, a bending standing wave was excited in the cylinder by the longitudinal vibration of transducer (*V_L_* = 100 V, *V_B_* = 0 V), and the vibration amplitude was gained. Then, the other bending standing was excited by the bending vibration of transducer (*V_B_* = 100 V, *V_L_* = 0 V), and the vibration amplitude wave was gained too. The results indicate that, in the two standing waves, the vibration amplitudes of wave loops are inconsistent. The main reason caused this inconsistence is the coupling effect between the cylinder and transducer, which results in the deformation of vibration mode shape of cylinder. Two mean values of vibration amplitudes of wave loops were taken to represent two standing waves, and results indicate that they are very close to each other. Thus, in the following analysis for traveling wave, *V_B_* and *V_L_* were set equal with each other.

**Figure 6:**
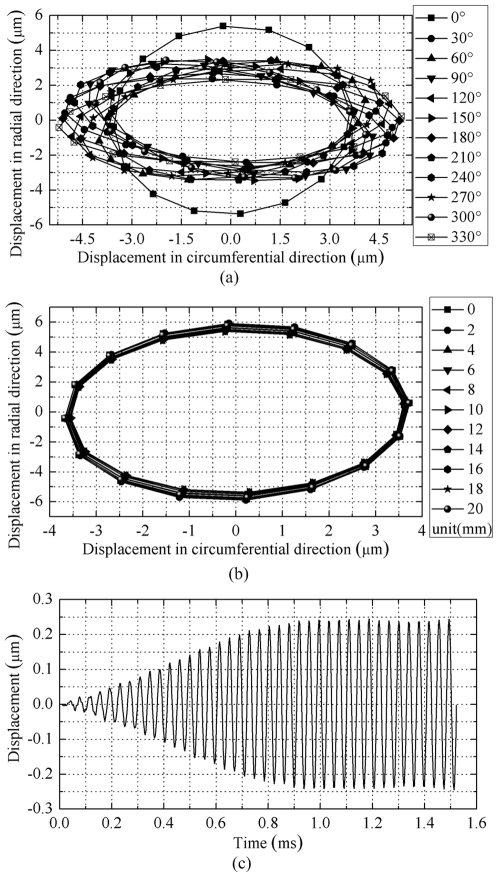
Motion trajectories and vibration amplitudes of nodes on the teeth. (a) Motion trajectories of nodes on evenly distributed teeth, and the angles in the legend are the angle positions of teeth relative to the central plane of transducer, (b) Motion trajectories of nodes on the same bus bar, and the lengths in the legend are the distances relative to the endpoint of bus bar, (c) The displacement of node in axial direction.

Finally, transient analysis was accomplished to test the vibration characteristics of stator for traveling wave excitation (*V_B_* = 200 V, *V_L_* = 200 V). The displacements of nodes on the teeth were extracted. Firstly, twelve nodes at the center of the inner surface of twelve evenly distributed teeth were selected, and the displacements on circumferential and radial direction were extracted. Fig. 6(a) shows the motion trajectories of selected nodes in the last simulation period. Then, eleven nodes on the same bus bar were selected, and Fig. 6(b) shows the motion trajectories. At last, one node on the side surface of tooth was selected, and Fig. 6(c) shows the displacement in axial direction in the whole simulation periods.

## Results

Fig. 6(a) indicates that the motion trajectories of selected nodes are ellipses, which is coincident with the actuating mechanism of traveling wave USM. But the vibration amplitudes are inconsistent with each other, which indicates the wave excited in the cylinder is not an ideal traveling wave. The main reason is the deformation of vibration mode shapes of cylinder. Previous simulation results have proved that, in the two standing waves, the vibration amplitudes of wave loops are inconsistent. Thus, the standing waves can not satisfy the condition for traveling wave very well (the amplitudes of two bending standing waves should be equal). The node on the teeth between the cantilevers has the maximum vibration amplitude in radial direction, while the motion trajectories of other nodes have better consistence. This inconsistency results in difference between the driving forces of teeth. There is 0.252 kHz difference between the resonance frequencies of stator and cylinder. The decreasing of this difference can improve the vibration mode shape of the standing waves and obtain more consistent vibration amplitudes.

Fig. 6(b) shows that the motion trajectories of nodes on the same bus bar have good consistence. Thus, all the particles on the inner surface of one tooth have nearly the same driving effect on the rotor.

Fig. 6(c) shows that the nodes on the teeth have periodic vibration in axial direction, which indicates the objective motions of nodes on the teeth are three-dimensional vibrations. But, compared with the vibrations in circumferential and radial direction, the vibration in axial direction is minute.

## Discussion

A cylindrical type traveling wave ultrasonic motor using cantilever type composite transducer was proposed in this paper. In this new design, a single transducer can excite a flexural traveling wave in the cylinder. The actuating mechanism of proposed motor was analyzed. The stator was designed and analyzed with FEM. The simulation results indicated that the motion trajectories of nodes on the teeth are nearly ellipses, which verify the feasibility of proposed design. But, the wave excited in the cylinder is not an ideal traveling wave, and the vibration amplitudes of teeth are inconsistent. The distortion of traveling wave is generated by the deformation of bending vibration mode of cylinder, which is caused by the coupling effect between the cylinder and transducer. Analysis results also prove that the objective motions of nodes on the teeth are three-dimensional vibrations. But, the vibration in axial direction is minute compared with the vibrations in circumferential and radial direction. The results of this paper verify the theoretical feasibility of proposed design and provide instructions for the development of proposed motor.
